# The Second-Agent Effect: Communicative Gestures Increase the Likelihood of Perceiving a Second Agent

**DOI:** 10.1371/journal.pone.0022650

**Published:** 2011-07-28

**Authors:** Valeria Manera, Marco Del Giudice, Bruno G. Bara, Karl Verfaillie, Cristina Becchio

**Affiliations:** 1 Center for Cognitive Science, Department of Psychology, University of Torino, Torino, Italy; 2 Laboratory of Experimental Psychology, Department of Psychology, Katholieke Universiteit Leuven, Leuven, Belgium; Royal Holloway, University of London, United Kingdom

## Abstract

**Background:**

Beyond providing cues about an agent's intention, communicative actions convey information about the presence of a second agent towards whom the action is directed (second-agent information). In two psychophysical studies we investigated whether the perceptual system makes use of this information to infer the presence of a second agent when dealing with impoverished and/or noisy sensory input.

**Methodology/Principal Findings:**

Participants observed point-light displays of two agents (A and B) performing separate actions. In the Communicative condition, agent B's action was performed in response to a communicative gesture by agent A. In the Individual condition, agent A's communicative action was replaced with a non-communicative action. Participants performed a simultaneous masking yes-no task, in which they were asked to detect the presence of agent B. In Experiment 1, we investigated whether criterion *c* was lowered in the Communicative condition compared to the Individual condition, thus reflecting a variation in perceptual expectations. In Experiment 2, we manipulated the congruence between A's communicative gesture and B's response, to ascertain whether the lowering of *c* in the Communicative condition reflected a truly perceptual effect. Results demonstrate that information extracted from communicative gestures influences the concurrent processing of biological motion by prompting perception of a second agent (second-agent effect).

**Conclusions/Significance:**

We propose that this finding is best explained within a Bayesian framework, which gives a powerful rationale for the pervasive role of prior expectations in visual perception.

## Introduction

Communicative gestures always presuppose the presence of a second agent [1], [2], [3]. Consider for example the gesture of pointing: This referential gesture works by directing the attention of another agent to some location in the surrounding environment. Even if no other person is visible in the scene, an observer watching an agent performing such a gesture would assume that there is a second person towards whom the gesture is directed [4]. This grants communicative gestures a special informational status: Beyond providing information about the actor, they also convey information about the presence of an interaction partner towards whom the action is directed (second-agent information).

The present study aimed at investigating to what extent, in the presence of impoverished and/or noisy visual information, the perceptual system makes use of this information to infer the presence of a second agent. Using a simultaneous masking detection task, Manera, Becchio, Schouten, Bara, and Verfaillie [5] demonstrated that observing a communicative gesture facilitates visual discrimination of a second agent. Participants observed point-light displays of two agents (A and B) performing separate actions. In the communicative condition, the action of agent B was performed in response to a communicative gesture performed by agent A. In the individual condition, agent A's communicative action was replaced with a non-communicative action. Results (in a two-alternative forced choice task) showed that observing the communicative gesture performed by agent A enhanced visual detection of agent B, embedded in a noise mask of moving point lights. These findings suggest that second-agent information facilitates perception of another agent by allowing observers to predict the other person's action. Put differently, in the context of a communicative interaction, the action of one agent can serve as a predictor of the other agent's action. But what happens when no other agent is in fact present? Are observers prompted to infer the presence of a second agent even when no agent is actually there?

In Bayesian terms, the function of the perceptual system is to build a plausible model of the world by optimally integrating currently available sensory evidence with expectations about the state of the external environment (*priors*, [6], [7]). There is convincing evidence that low-level perceptual activity can be modulated by higher-level cognitive factors [8] and that expectations – either induced by instructions or based on the statistical distribution of previous sensory inputs - can strongly affect the contents of visual awareness [9], [10]. Critically, reliance on prior expectations has been shown to vary according to the type of observed action. For simple goal directed actions such as lifting an arm or pressing a button, perceptual judgment primarily relies on the external visual input. For actions directed at more complex goals (e.g., actions requiring the execution of a combination of basic actions) prior expectations exert a stronger influence, to the detriment of the available sensory information. A similar over-reliance on priors is reported when observed actions fit into a context of social interaction: Prior expectations are favored over visual information [11]. In this framework, observing a communicative action should influence perception by increasing the prior probability that a second-agent is present in the scene. This updating of prior expectations can be expected to bias observer's performance especially when stimulus-driven processing is made more difficult (e.g., by simultaneous masking). Indeed, the less reliable the input, the more perception is influenced by the prior. As a result, observers might be induced to perceive a second agent, irrespectively of whether a second agent is in fact present or not.

Signal detection theory (SDT) provides a suitable tool to investigate this process [12], [13]. In SDT, participants' correct responses and errors in a detection task are used to estimate two parameters, the *sensitivity* (*d′*) and the *response criterion* (*c*). Sensitivity is a measure of the individual's ability to discriminate between signal and noise (e.g., between presence and absence of a second agent in an animated clip); higher values of *d′* (ranging from 0 to +∞) indicate better discrimination ability. The response criterion is a complex parameter, reflecting both the expected likelihood of the signal being present in the stimulus and the decision process involved in the response. Lower values of *c* (ranging from −∞ to +∞) may indicate (a) a higher expected probability that the signal will be present, and/or (b) a more liberal decision threshold, requiring a lower degree of certainty before a positive response is given. If observing a communicative gesture increases the probability of perceiving a second agent - whether or not a second agent is in fact present - then a lower response criterion should be observed in visual detection of a human agent. Specifically, let *P(S)* be the expected (prior) probability of signal (i.e., a second agent is present), and *P(N)* be the expected (prior) probability of noise (i.e., there is no second agent); the optimal response criterion *c* ^*^ is
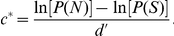



Thus, when the prior probability of a second agent being present increases, the optimal response criterion will decrease accordingly. Here, we tested this hypothesis in two psychophysical experiments.

### Overview of Experiments and Results

#### Experiment 1

In Experiment 1, participants viewed point-light stimuli of two human figures belonging to two experimental conditions. Stimuli in the *Communicative condition* displayed a communicative interaction between two agents, with agent A performing a communicative gesture towards a second agent (B), who responded accordingly (e.g., A asks B to squat down, B squats down). Stimuli in the *Individual condition* were assembled by replacing agent A's communicative action with a non-communicative action (e.g., A turns, B squats down). Participants performed a simultaneous masking yes-no task, in which they were asked to discriminate between signal trials (containing agent B), and noise trials (not containing agent B).

The results of Experiment 1 showed that the criterion was lower in the Communicative condition than in the Individual condition. This suggests that observing a communicative gesture increased the probability of perceiving a second agent, regardless of whether a second agent was in fact present or not. However, an alternative explanation might be that observers were simply more inclined to report a signal in the Communicative condition compared to the Individual condition. If this were the case, the lowered criterion in the Communicative condition might reflect mere response bias. A second experiment was designed to resolve this issue, by manipulating the congruency of the two agents' actions in the Communicative condition.

#### Experiment 2

The aim of Experiment 2 was to ascertain whether the lowered criterion in the Communicative condition in Experiment 1 reflected a perceptual effect (i.e., an increased likelihood of perceiving a second agent) rather than mere response bias (i.e., an increased tendency to respond “yes”). To this end, we extended Experiment 1 by adding a third experimental condition, i.e. a *Communicative-incongruent condition*, in which agent B's response (e.g., B squats down) did not match A's communicative gesture (e.g., A asks B to come closer). If the lowered criterion reflected mere response bias, no difference in *c* should be observed between the Communicative-congruent condition and the Communicative-incongruent condition. In contrast, if observers were truly more likely to perceive a second agent, we would expect a significant decrease in *c* in the Communicative-congruent condition compared to the Communicative-incongruent condition. This is because in the congruent condition, but not in the incongruent condition, the actions of agent A can be used to anticipate the actions of agent B. We expected this to occur in both signal and noise trials, as the noise trials contain elements of the corresponding actions of agent B (see Experiment 1: Method).

Experiment 2 replicated the finding of Experiment 1, suggesting that observers were more likely to see a second agent when presented with Communicative-congruent actions compared with individual actions. Also, the criterion was significantly lower in the Communicative-congruent condition compared to the Communicative-incongruent condition; this rules out the possibility that the second-agent effect only reflects changes in response bias. Indeed, if our results were fully accounted for by response bias, the same lowering of response criterion should have been observed in both communicative conditions. The data from a post-experiment questionnaire further supported a perceptual interpretation, by showing that, when incorrectly reporting the presence of agent B, participants reported perceiving a human figure (or part of it).

## Methods

### Experiment 1

#### Participants

Twenty-three undergraduate and graduate students from the University of Turin (8 male and 15 female, mean age = 26.5 years, age range 21–34) volunteered to take part in the experiment. All had normal or corrected-to-normal vision, had provided informed written consent and were naïve with respect to the purpose of the study. The study was approved by the Ethical Committee of the Faculty of Psychology of the University of Turin and was conducted in accordance with the ethical standards laid down in the 1964 Declaration of Helsinki.

#### Stimuli

Stimuli consisted of two point-light figures with 13 markers indicating the centre of the major joints of a person (head, shoulders, elbows, wrists, hips, knees, and feet). 10 point-light stimuli were employed, 5 belonging to the Communicative condition and 5 belonging to the Individual condition. Stimuli in the Communicative condition displayed a communicative interaction between two agents, with agent A performing a communicative gesture towards a second agent (B), who responded accordingly. These stimuli were selected from the Communicative Interaction Database (CID [14]) and included: ‘Get down’, ‘Pick it up’, ‘Look at that (ceiling)’, ‘Help yourself’, and ‘Sit down’. Stimuli in the Individual condition were assembled by replacing agent A's communicative action with a non-communicative action executed with the same onset and duration (‘Turn’, ‘Jump’, ‘Sneeze’, ‘Lateral step’, and ‘Drink’). In both the Communicative and the Individual conditions, agent B's action (e.g., ‘picking something up’) was always coupled with a fixed action by agent A (‘pointing to something to be picked up’ in the Communicative condition; ‘jumping’ in the Individual condition).

Stimuli were constructed combining motion capture techniques and animation software [15]. For the Communicative condition the actions of the two actors were captured simultaneously, in order to guarantee that B's response matched A's communicative gesture in all respects (e.g. timing, position, kinematics). A and B were always visible, but the onset of A's action always preceded that of B's action. For the Individual condition A's action was captured while the actor was acting alone and was then coupled with B's action, so as to maintain the same temporal structure as in the communicative interaction (i.e., A's action had the same onset and duration as in the Communicative condition). Stimulus duration ranged from 3,600 to 8,200 ms. In both the Communicative and the Individual conditions, agent A and agent B remained approximately at a constant distance from the centre of the screen for the whole duration of the action and never physically touched one another. In all action stimuli (in both the Individual and in the Communicative conditions), the agents always faced each other. Previous research employing the present stimuli has shown that communicative and individual actions can be clearly distinguished, and that gestures can be easily identified by untrained observers (see Manera et al. [14] for a description of stimulus recognisability).

#### Apparatus and procedure

Stimuli were presented on a 15.4-inch WXGA screen (display resolution: 1280×800; refresh rate: 60 Hz) using MatLab (7.1 version) software. Viewing distance was 60 cm. Stimuli were black against a grey background, and were rendered from a three-quarter view (corresponding to the 125° reference orientation used in the CID). The visual angle between the points attached to the head and the foot was about 7.15 deg and individual points subtended approximately 0.14 deg each. Participants were tested individually in a dimly lit room.

**Figure 1:**
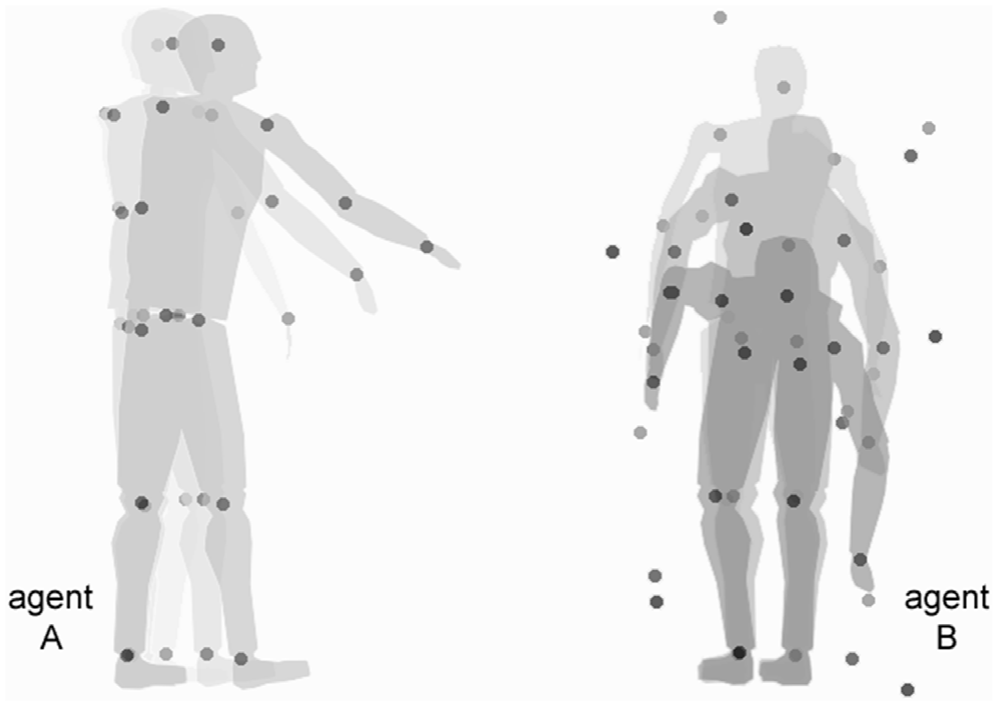
Example of a communicative signal trial. Agent A points to an object to be picked up; agent B bends down and picks it up. B was presented using limited-lifetime technique (6 signal dots) and masked with temporally scrambled noise dots. The noise level displayed is the minimum allowed in the experiment (5 noise dots). To provide a static depiction of the animated sequence, dots extracted from 3 different frames are superimposed and simultaneously represented; the silhouette depicting the human form was not visible in the stimulus display.

As we were interested in variations in criterion, we did not use a forced-choice task as in Manera et al. [5], but a yes-no task [16]. Participants were asked to distinguish between two kind of trials: signal trials (containing agent B) and noise trials (not containing agent B). In *signal trials*, B's actions were displayed using a limited lifetime technique and masked with limited lifetime noise dots [17], [18]. Each signal dot was presented for a fixed duration (200 ms) at one of the 13 possible locations, then disappeared and reappeared at another randomly chosen location. Six signal dots per frame were shown. Dot appearance and disappearance were asynchronous across dots in order to avoid motion transients from simultaneous transitions of all sampling dots. Noise dots had the same trajectories, size, and duration as the signal dots, but were temporally and spatially scrambled (they appeared in a region sustaining a visual angle of approximately 8.6 deg horizontally and 14.3 deg vertically). The number of noise dots was adjusted individually for each participant in a training session (see below). In *noise trials*, agent B was substituted by limited lifetime scrambled dots obtained by temporally scrambling the corresponding signal action. Noise dots were also added so as to obtain the same number of dots as displayed in the signal trials. Because the position and motion of the dots in noise trials equaled (on average) those of signal trials, noise trials were perceptually similar to the corresponding signal trials [17]. In both signal and noise trials, A was neither limited lifetime nor masked (see Figure 1).

Participants were instructed to look at each stimulus and to decide whether it was a signal trial or a noise trial. Responses were given by pressing one of two keys on a keyboard. Participants were informed that there were two conditions (Individual vs. Communicative) and that, in the Communicative condition, the actions of A and B were semantically related. This was meant to ensure that participants devoted proper attention to agent A's actions in both conditions. In order to minimize response bias, participants were also informed that the probability of signal or noise was the same in both conditions. Each participant completed four 30-trial blocks: 10 actions (5 communicative+5 individual) by 2 types of trial (signal or noise) by 6 repetitions. Each block consisted of (signal and noise) trials of both conditions presented in randomized order. Blocks lasted approximately seven minutes each and were separated by a rest period of two minutes. Accuracy feedback was provided after each block.

#### Training session

Stimuli consisted in five actions performed by a single agent, masked with five levels of noise (5, 15, 25, 35 or 45 noise dots). Training actions were selected from the CID and included ‘raising arms’, ‘doing aerobics’, ‘picking something up’, ‘standing up’ and ‘turning’. Each participant completed four blocks of 25 trials (five actions by five noise levels and by two kinds of trials –signal/noise – by two repetitions). Trials in each block were presented in randomized order. Individual noise levels were determined by fitting a cumulative Gaussian function to the proportion of correct responses and determining the 75% threshold. The minimum noise level allowed was five noise dots. Individual noise levels determined in the preliminary session ranged from 5 to 26 dots (M = 8.7, SD = 6.2).

#### Data Analysis

Following administration, each response was coded as correct or incorrect. For both the Communicative and the Individual condition, a correct response was scored for a “yes” response on signal trials and for a “no” response on noise trials. In order to compare performance in the Communicative and Individual condition, we also extracted Signal Detection Theory parameters. For each participant we calculated the number of hits (“signal” responses on signal trials) and false alarms (“signal” responses on noise trials) in the two experimental conditions. For each condition, the hit rate (H) was calculated by dividing the number of hits by the total number of signal trials (N = 30), and the false alarm rate (F) was calculated by dividing the number of false alarms by the total number of noise trials (N = 30). Following the standard procedure, rates of 0 were replaced with 0.5/N, and rates of 1 were replaced with (N−0.5)/ N, where N is the number of signal or noise trials [19]. Criterion (c) and sensitivity (d′) parameters were extracted as follows [16]:
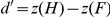


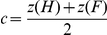
where the function *z* is the inverse of the normal distribution function.

### Experiment 2

#### Participants

Thirty-three undergraduate and graduate students from the University of Turin (8 male and 25 female, mean age = 20.4 years, age range 18–29) volunteered to take part in the experiment. All had normal or corrected-to-normal vision, had provided informed consent and were naïve with respect to the purpose of the study. None of Experiment 2 participants had participated in Experiment 1. The study was approved by the Ethical Committee of the Faculty of Psychology of the University of Turin and was conducted in accordance with the ethical standards laid down in the 1964 Declaration of Helsinki.

#### Stimuli

Stimuli consisted of two point-light figures, and belonged to 3 experimental conditions:

1. *Communicative-congruent condition*. As in Experiment 1, stimuli displayed a communicative interaction between two agents, with agent A performing a communicative gesture towards agent B, who responded accordingly (e.g., A asks B to squat down, B squats down).
2. *Communicative-incongruent condition*. Stimuli were assembled by replacing agent A's communicative action with a different communicative action executed with the same timing (communicative incongruent actions selected from the CID: ‘Come closer’, ‘Imitate me’, ‘Move it’, ‘Move over’, and ‘No’). As a result, B's response did not match A's request(e.g., A asks B to come closer, B squats down).
3. *Individual condition*. As in Experiment 1, stimuli were assembled by replacing agent A's communicative action with a non-communicative action executed with the same timing (e.g., A turns around, B squats down).

#### Apparatus and procedure

Apparatus, procedure, and data analysis were the same as in Experiment 1. Participants were informed that stimuli could be communicative or individual; they were further told that, in the Communicative conditions, the actions of A and B might or might not be semantically related. Participants completed four blocks of 45 trials: 15 actions (5 Communicative-congruent+5 Communicative-incongruent+5 Individual) by 2 types of trial (signal or noise) by 6 repetitions.

#### Post-experiment questionnaire

After the experimental session, participants were shown each stimulus again and asked to indicate a) whether the action of agent A was communicative or individual, and b) whether agent B was present or not. When participants reported the presence of agent B, they were asked to specify whether they a) had been guessing, b) had seen a human figure (or some of its body parts) but did not understand what she was doing, or c) had seen a human figure performing a specific action.

## Results and Discussion

### Experiment 1

The mean proportion of correct answers was .71 (score range = .53–.97), suggesting that the threshold estimate calculated in the training session had been sufficiently accurate for most of participants (Communicative condition: *M* = .72, score range = .55–.95; Individual condition: *M* = .71, score range = .53–.97). The “Pick it up” action was the most difficult to detect (*M* = .63), followed by “Squat down” (*M* = .70), “Look at that-ceiling” (*M* = .71), and “Help yourself” (*M* = .74). The most easily detected action was “Sit down” (*M* = .79).

**Figure 2:**
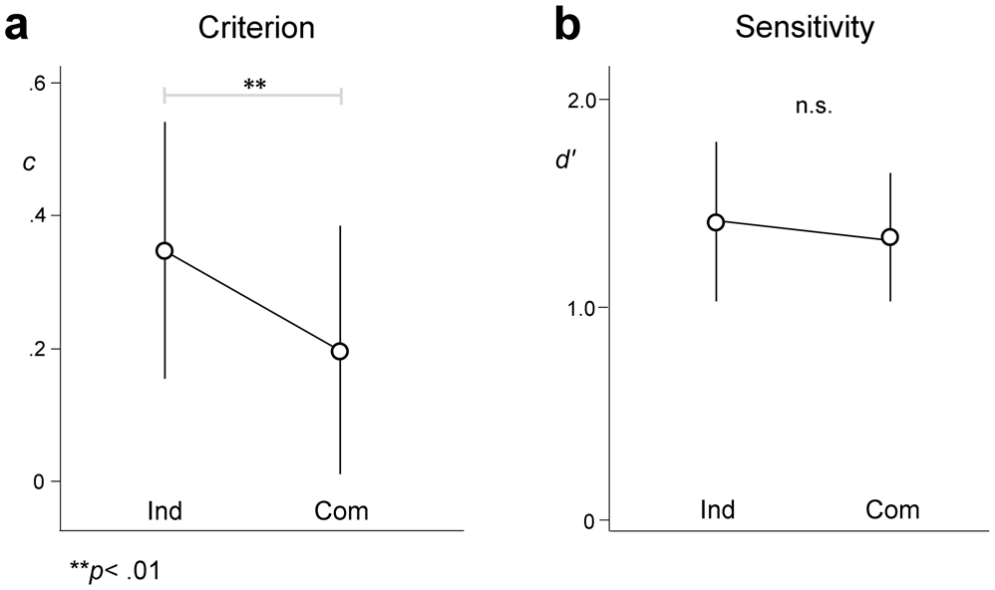
SDT parameters for the two experimental conditions. Error bars are 95% confidence intervals.

In both the Communicative and the Individual condition, participants displayed a conservative detection threshold, as indicated by the positive mean values of *c.* Criterion values ranged from −.80 to 1.31 (*M* = .20; *SD* = .43) in the Communicative condition and from −.64 to 1.44 (*M* = .35; *SD* = .45) in the Individual condition. Sensitivity values ranged from .26 to 3.36 (*M* = 1.34; *SD* = .83) in the Communicative condition and from .17 to 3.80 (*M* = 1.41; *SD* = 1) in the Individual condition (Figure 2). Within-subjects ANOVA revealed that, in the Communicative condition, the response criterion was significantly lower than in the Individual condition (*F*_(1,22)_ = 9.55; *p* = .005; Figure 2a). No significant difference in sensitivity was found (*F*_(1,22)_ = .43; *p* = .517; Figure 2b).

### Experiment 2

**Figure 3:**
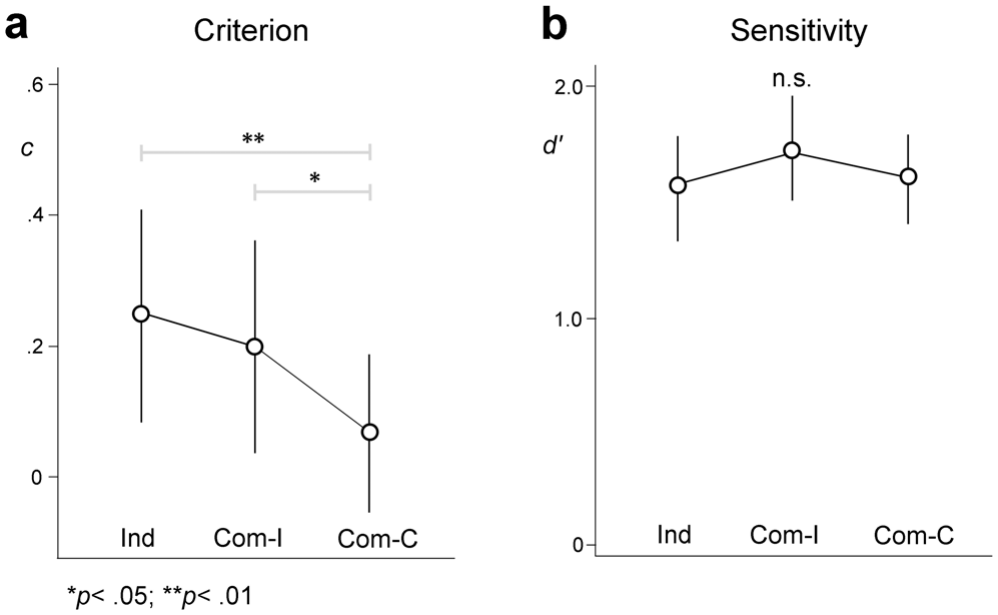
SDT parameters for the three experimental conditions. Error bars are 95% confidence intervals.

The mean proportion of correct responses was .76 (score range = .48–.92). The “Pick it up” action was the most difficult to detect (*M* = .69), followed by “Help yourself” (*M* = .75), “Look at that ceiling” (*M* = .77), and “Squat down” (*M* = .79). The most easily detected action was “Sit down” (*M* = .80). For each participant we extracted criterion (*c*) and sensitivity (*d′*) parameters in the three experimental conditions. In all conditions participants showed a conservative detection threshold, as indicated by the positive mean values of the criterion parameter *c*. Criterion values ranged from −.62 to .93 (*M* = .07; *SD* = .34) in the Communicative-congruent condition, from −.70 to 1.23 (*M* = .20; *SD* = .45) in the Communicative-incongruent condition, and from −.61 to 1.05 (*M* = .24; *SD* = .47) in the Individual condition. Sensitivity values ranged from .73 to 2.95 (*M* = 1.59; *SD* = .58) in the Communicative-congruent condition, from −.08 to 3.09 (*M* = 1.73; *SD* = .66) in the Communicative-incongruent condition, and from −.09 to 2.68 (*M* = 1.55; *SD* = .68) in the Individual condition (Figure 3).

In order to compare criterion and sensitivity among the three experimental conditions we performed separate ANOVAs with condition (Communicative-congruent, Communicative-incongruent, and Individual) as the within-subject factor. For the criterion parameter, ANOVA revealed a significant effect of condition (*F*_2,64)_ = 7.08; *p* = .002), with *c* linearly decreasing from the Communicative-congruent condition to the Communicative-incongruent condition to the Individual condition (linear contrast: *F*_(1,32)_ = 15.33; *p*<.001; Figure 3a). Post-hoc comparisons (with Bonferroni correction) revealed that *c* was significantly lower in the Communicative-congruent condition compared to both the Communicative-incongruent condition (*p* = .034) and the Individual condition (*p* = .001). No significant difference between the Communicative-incongruent condition and the Individual condition was found (*p* = 1.000). For the sensitivity parameter, no significant effect was found in the ANOVA (*F*_(2,64)_ = 2.12; *p* = .128). Furthermore, post-hoc comparisons revealed no significant difference between the three experimental conditions (*p*_s_ ranging from .185 to 1.000; see Figure 3b).

In the post-experiment questionnaire, participants were accurate in distinguishing between Communicative and Individual stimuli; the mean proportion of correct responses was .82 (Communicative-congruent = .81; Communicative-incongruent = .81; Individual = .83). Communicative-congruent and Communicative-incongruent stimuli were classified as communicative equally often (*t*_(32)_ = −.08, *p* = .931). Concerning agent B detection, the proportion of correct responses was .76. False alarms (i.e., signal reported when no signal was present; n = 105) amounted to 45% of the total errors, and were evenly distributed across experimental conditions. The great majority of participants committing false alarms reported having seen a human figure performing a specific action (44% of the false alarms), or having seen a human figure (or some of its body parts) performing an action they could not understand (52% of false alarms). Only a negligible percentage of participants reported guessing (4% of false alarms).

It is worth noticing that, even though no significant difference in criterion was found between the Communicative-incongruent condition and the Individual condition, the response criterion showed a significant linear decrease, being highest in the Individual condition, lower in the Communicative-incongruent condition, and lowest in the Communicative-congruent condition. This finding is consistent with a number of possible explanations. For example, it is possible that response bias made a minor contribution to changes in *c*; also, this pattern of results may reflect the combination of two perceptual effects: a specific effect (B's action is predicted based on A's communicative action) and a generic effect (the presence, but not the specific action, of B is predicted based on the communicative nature of A's action). Future studies will be necessary to decide between these different explanations. First, functional MRI studies may help disentangling perceptual and non-perceptual aspects in the second-agent effect. Perception of biological motion has been consistently related to regions along the superior temporal sulcus, the motion-sensitive region MT, the parietal cortex, and other regions in visual cortex [20], [21], as well as to premotor areas in frontal cortex [22]. If the second agent effect is perceptual in nature, lowering of criterion in the communicative condition might be expected to modulate activity within these regions. Second, experiments in which both agents are masked and participants are not aware that they will observe communicative actions might help clarifying to what extent conscious attribution of communicative intention is necessary to produce a second-agent effect.

### General Discussion

Previous research has shown that people can accurately distinguish between communicative and individual actions under point-light conditions [14] and that observing a communicative gesture improves detection of the presence of a second agent [5]. In this study, we demonstrated for the first time that information extracted from communicative gestures influences the concurrent processing of biological motion by prompting perception of a second agent, regardless of whether a second agent was in fact present or not (second-agent effect). This effect does not merely depend on observing a generic action performed by another agent (as we found reliable differences between the Communicative and Individual condition), nor on the observation of a communicative gesture *per se* (as we found differences between the Communicative-congruent and Communicative-incongruent condition). Rather, the results point to a *specific* effect based on interpersonal predictive coding, i.e. perception of the communicative gesture of one agent is used to predict the other agent's action (creating prior expectations concerning the presence of the second agent).

Perception of others' action is not simply a post-hoc reconstruction of the visual input, but an intrinsically predictive activity [23]. Predicting what kind of actions others will perform, as well as when and where others will act is essential for successful interaction [24]. If humans merely reacted to what they saw others doing, they could not anticipate their goals and intentions. Most importantly, they could never achieve the smooth and fast coordination needed to actively and directly online interact with others [25].

The second agent effect provides a notable demonstration of the impact that prior expectations can have on the processing of others' actions in social contexts: expectations derived from interpersonal predictive coding can be so strong as to generate the illusion to see an agent even when no such agent is in fact present, a sort of “Bayesian ghost.”
